# Increasing the Productivity of Glycopeptides Analysis by Using Higher-Energy Collision Dissociation-Accurate Mass-Product-Dependent Electron Transfer Dissociation

**DOI:** 10.1155/2012/560391

**Published:** 2012-05-30

**Authors:** Julian Saba, Sucharita Dutta, Eric Hemenway, Rosa Viner

**Affiliations:** Thermo Fisher Scientific, San Jose, CA 95134, USA

## Abstract

Currently, glycans are attracting attention from the scientific community as potential biomarkers or as posttranslational modifications (PTMs) of therapeutic proteins. However, structural characterization of glycoproteins and glycopeptides remains analytically challenging. Here, we report on the implementation of a novel acquisition strategy termed higher-energy collision dissociation-accurate mass-product-dependent electron transfer dissociation (HCD-PD-ETD) on a hybrid linear ion trap-orbitrap mass spectrometer. This acquisition strategy uses the complementary fragmentations of ETD and HCD for glycopeptides analysis in an intelligent fashion. Furthermore, the approach minimizes user input for optimizing instrumental parameters and enables straightforward detection of glycopeptides. ETD spectra are only acquired when glycan oxonium ions from MS/MS HCD are detected. The advantage of this approach is that it streamlines data analysis and improves dynamic range and duty cycle. Here, we present the benefits of HCD-PD-ETD relative to the traditional alternating HCD/ETD for a trainer set containing twelve-protein mixture with two glycoproteins: human serotransferrin, ovalbumin and contaminations of two other: bovine alpha 1 acid glycoprotein (bAGP) and bovine fetuin.

## 1. Introduction

Glycosylation is an important PTM that plays crucial roles in various biochemical processes, ranging from mediation of interactions between cells to defining cellular identities within complex tissues [1, 2]. In addition, glycan structures are unique to the proteins which they are attached to and to the site of attachment and, thus, play crucial roles in controlling the activities of the proteins. Many glycans, also, show disease-related expression level changes [3, 4]. For example, changes in glycosylation patterns have been used as a means to monitor progression of cancer [3–6]. In many instances, it is essential to characterize the exact glycan structure and site of glycosylation to better understand the protein-mediated interaction that these glycans undergo.

Mass spectrometry (MS) has emerged as one of the most powerful tools for proteomics due to its sensitivity of detection and its ability to analyze complex mixtures derived from a variety of organisms and cell lines. However, structural characterization of glycoproteins/glycopeptides remains analytically challenging due to the reliance on traditional acquisition strategies and MS/MS fragmentation techniques.

Conventional proteomics has benefitted tremendously from collision-activated dissociation (CAD) due to the ease of implementation of the technique on most commercial mass spectrometers and the abundant peptide bond cleavages that this technique generates, resulting in large number of peptides and proteins identified. Unfortunately for glycoproteomics, CAD does not provide the necessary fragment ions to thoroughly characterize intact glycopeptide structures [7]. Depending upon the mass spectrometers used, CAD provides varying degrees of structural information. For example, low-energy CAD on most mass spectrometers predominantly generates glycosidic bond cleavages with minimal fragmentation occurring along the peptide backbone [8–11]. Additionally, cleavages also tend to occur between the peptide-glycan bond, resulting in loss of information about the site of glycosylation. The increase of collision energy can result in more efficient fragmentation of the peptide backbone, but this strategy can result in mixed MS/MS spectra where both the glycan and peptide fragment ions are present in the same spectra, complicating spectral interpretation [12]. Regardless of whether high- or low-energy CAD is employed, fragmentation of the peptide-glycan bond still occurs limiting the ability to derive information about the site of glycosylation. Majority of the current approaches have foregone the strategy of examining intact glycopeptides and have focused on obtaining partial information, such as sequencing peptide backbone and identifying sites of glycosylation. For example, the use of *N*-glycosidase F or A (PNGase F/A) enzymes results in the removal of glycans and conversion of asparagines, the site of glycosylation to aspartic acid. This conversion process can then be monitored by high-resolution MS due to a mass shift of 0.9840 Da to identify the site of glycosylation. Additionally, one can increase the confidence in the glycosylation site assignment by incorporation of stable isotope labeling by performing PNGaseF/A digestion in the presence of H_2_O^18^. Such approach involves the release of glycans by a deamidation reaction, and the incorporation of H_2_O^18^ will lead to a mass shift of 2.9890 Da on the asparagine residue. But, studies have shown that these types of chemical deamidations can occur spontaneously during sample preparation for release of glycans in the presence and absence of H_2_O^18^ leading to number of false positives [13–17]. These issues underline the importance of intact glycopeptides structural analysis, and only this approach enables comprehensive structural characterization. Though CAD is limiting for glycopeptides analysis, alternative fragmentation techniques such as electron-capture dissociation (ECD) [18] and ETD [19, 20] are better suited for glycopeptides analysis due to their nonergodic type of dissociation. These dissociation techniques induce extensive fragmentation of the peptide backbone enabling sequencing of the peptide but preserving glycans on the peptide backbone allowing unambiguous assignment of the glycosylation sites, thus providing complementary information to CAD.

Previously, several studies have demonstrated the utility of combining CAD and ETD fragmentation for glycopeptides characterization [19–22]. For example, Catalina et al. have performed detailed analysis of horse peroxidase using alternating CID, ETD fragmentation [20]. We have shown in the past the importance of combining ETD and CAD, specifically HCD, for full structural characterization of glycopeptides [22]. However, all of these studies have used both types of fragmentation in an unselective fashion. Here, we expand on this approach to report a novel acquisition strategy termed HCD-PD-ETD that enables on the fly identification of glycopeptides and improves overall productivity of glycopeptides analysis. In this approach, the LTQ Orbitrap Velos acquires HCD spectra in a data-dependent fashion. The instrument identifies glycan oxonium ions on the fly in the HCD spectra and triggers ETD spectra on the glycopeptides precursor only. The advantage of this approach is that it streamlines data analysis and improves dynamic range and duty cycle. The benefits of this novel acquisition strategy are examined for a trainer set containing twelve-protein mixture with two glycoproteins and contaminants of two others and compared against the traditional alternating HCD/ETD approach.

## 2. Materials and Methods

All protein and glycoprotein standards used in these experiments were purchased from Sigma-Aldrich Chemical Co. (St. Louis, MO, USA).

### 2.1. Protein Reduction, Alkylation, and Digestion

100 *μ*g of equal molar twelve-protein mixture (lysozyme, glyceraldehyde-3-phosphate dehydrogenase, beta-casein, cytochrome c, bovine serum albumin, ovalbumin, carbonic anhydrase, serotransferrin, alpha-lactalbumin, apo-myoglobulin, bAGP, and bovine fetuin) was dissolved in 100 *μ*L of 50 mM TrisHCl, pH 8.6/0.1% SDS buffer. This was followed by reduction with 5 *μ*L of 100 mM *tris*(2-carboxyethyl)phosphine (TCEP) and incubation at 37°C for 30 mins. The reduced twelve-protein mixture was then alkylated with 5 *μ*L of 375 mM iodoacetic acid for 60 minutes, protected from light. Upon alkylation, the twelve-protein mixture was acetone precipitated over night at −30°C. The acetone-precipitated twelve-protein mixture was dissolved in 100 *μ*L of 100 mM triethyl ammonium bicarbonate (TEAB) and digested at 37°C for 4 hours with 2.5 *μ*g of trypsin (1 : 40 enzyme : protein ratio).

### 2.2. TMT Labeling

Isobaric labeling was accomplished with TMT^6^ reagent (Thermo Scientific, Rockford, IL, USA) per manufacturer's suggestion. Briefly, the reagent was dissolved in 41 *μ*L of anhydrous acetonitrile and added to 100 *μ*g of tryptically digested twelve-protein mixture. The sample was then incubated at room temperature for 1 hour and quenched with the addition of 8 *μ*L of 5% hydroxylamine and concentrated to 10 *μ*L using Speed Vac (Thermo Scientific, Waltham, MA, USA).

### 2.3. LC/MS

**Figure 1:**
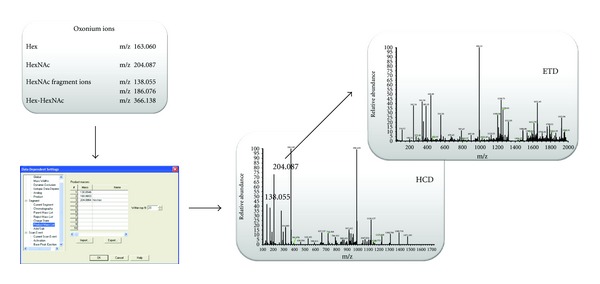
Schematic representation of HCD-PD-ETD acquisition method.

A Thermo Scientific EASY-nLC nano-HPLC system and Michrom Magic C18 spray tip 15 cm × 75 *μ*m i.d. column (Auburn, CA) were used. Gradient elution was performed from 5 to 45% ACN in 0.1% formic acid over 60 min at a flow rate of 300 nL/min. The samples were analyzed with a Thermo Scientific LTQ Orbitrap Velos hybrid mass spectrometer with ETD. The following MS and MS/MS settings were used: FT: MSn AGC Target = 5e4; MS/MS = 1 *μ*scans, 200 ms max ion time; MS = 400–2000 m/z, 60000 resolution at m/z 400, MS Target = 1e6; MS/MS = Top 10 data-dependent acquisition HCD product-dependent acquisition ion trap ETD (Figure 1), dynamic exclusion = repeat count 1, Duration 30 sec, exclusion duration 90 sec; HCD Parameters: collision energy = 35%; resolution 7500. MSn target ion trap = 1e4, 3 *μ*scans, 150 ms max ion time; ETD anion AGC target = 2e5, and charge-dependent ETD reaction time was used.

### 2.4. Data Processing

The prototype GlycoMaster (Bioinformatics Solution, Waterloo, ON, Canada) software was used for intact glycopeptides analysis. Searches were performed using a 5 ppm precursor ion tolerance and 0.8 Da ETD tolerance and 0.02 Da HCD tolerance, while allowing up to one missed cleavage. For peptide searches, carboxymethylation of cysteine residues (+58.005478 Da) was set as static modification. TMT^6^ tag on lysine residues and peptide N termini (+229.16293 Da) were set as variable modification. For glycan identification, the following possible monosaccharide combinations were searched: Hexose, HexNAc, Deoxyhexose, Neu5Gc, and Neu5Ac. Both O- and N-linked glycosylations were considered with H, Na, and K as potential adducts. To minimize on false hits for site of glycosylation, GlycoMaster considered N-linked glycosylation to occur at the amide residue of asparagine in a consensus sequence of Asn-Xaa-Ser/Thr (X can be any amino acid except proline) and O-linked glycosylation to occur at serine or threonine residues. All results were manually validated.

## 3. Results and Discussion

Current MS/MS acquisition strategies for glycopeptides analysis rely on acquiring MS/MS spectra for all precursors in a mass spectrum regardless of whether the precursor belongs to a glycopeptide. The resultant spectra are then interrogated after acquisition for characteristic glycan oxonium ions (which are dominant in the MS/MS CAD spectra) for detection of glycopeptides, and the corresponding spectra are then used in the identification [19–25]. The limitation of this approach is that both data acquisition and data interrogation are not performed as efficiently as possible. By triggering MS/MS spectra on all precursors, both duty cycle and dynamic range of analysis are decreased. Furthermore, postacquisition extraction of glycan oxonium ions requires additional tools to ascertain information about detected glycopeptides: their charge state and m/z. Further adding to the inefficiency of the current MS/MS acquisition strategies is that if a mass spectrometer with nominal mass accuracy is used, then more than one glycan oxonium ion must be extracted to minimize on false positives due to near mass isobaric ions [26]. However, in our approach, we employ HCD to generate glycan oxonium ions. The advantage of using HCD is that the generated fragment ions are measured within the orbitrap mass analyzer with high resolution and mass accuracy (HR/AM) allowing for unambiguous assignment of glycan oxonium ions [23]. Using orbitrap detection to our advantage, we have implanted a novel instrumental control within the hybrid linear ion trap-orbitrap mass spectrometer termed HCD-PD-ETD (Figure 1). This instrumental control enables the mass spectrometer to identify glycopeptides on the fly and acquire data in an intelligent fashion. In this approach, we specify certain glycan oxonium ions for the instruments to monitor within the HCD spectrum (up to ten different product ions can be specified). We typically use m/z 204.0864 HexNAc oxonium ion and its fragments 168.0653 and 138.0550 as diagnostic ions to be monitored in HCD fragmentation, and, by method definition, the monitored ions should be in the top 20–30 most intense fragment ions. However, if glycopeptides of interest do not contain terminal HexNAc, other diagnostic oxonium/product ions can be selected and collision energy optimized accordingly. The hybrid linear ion trap-orbitrap mass spectrometer equipped with ETD then acquires HCD spectra in a data-dependent fashion. Each HCD spectrum is interrogated by the instrument on the fly for the presence of diagnostic glycan oxonium (product) ions at ppm mass accuracy. If a diagnostic glycan oxonium (product) ion is detected, an ETD spectrum for the glycopeptide precursor is acquired. It should be noted that we can specify mass accuracy as low as 5 ppm for high selectivity. This novel acquisition strategy provides numerous advantages. Primarily, it increases overall productivity for MS analysis of glycopeptides by acquiring ETD spectra only when a glycopeptide is detected, enabling improvements in both duty cycle and dynamic range of analysis. Additionally, this approach improves data analysis by decreasing the rate of false positives, the overall file size, and the number of ETD spectra that are extrapolated to characterize glycopeptides.

**Figure 2:**
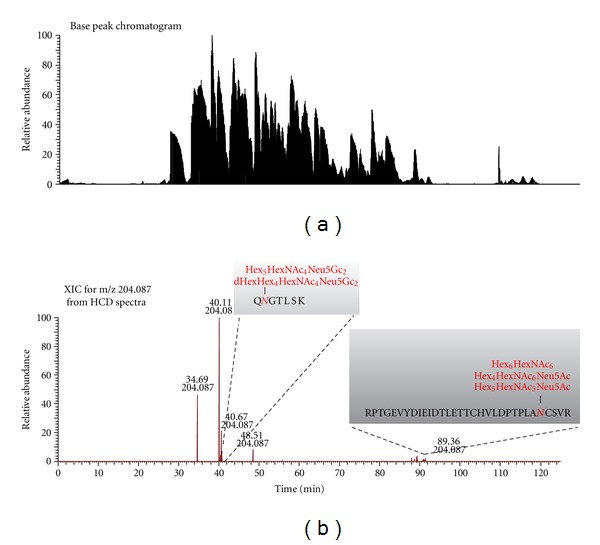
(a) Base peak chromatogram and (b) HCD XIC of HexNAc oxonium ion at m/z 204.087 of twelve-protein mixture digest by HCD-PD-ETD.

**Figure 3:**
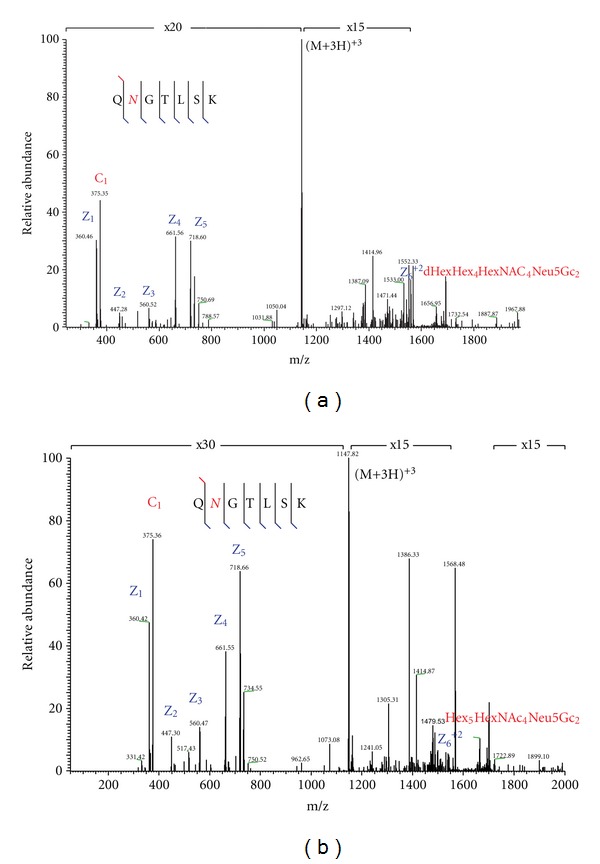
LC-MS ion trap ETD spectrum of bAGP N-linked glycopeptide T103–109 precursor at (a) m/z 1165.823 (3+) and (b) m/z 1148.174 (3+).

**Figure 4:**
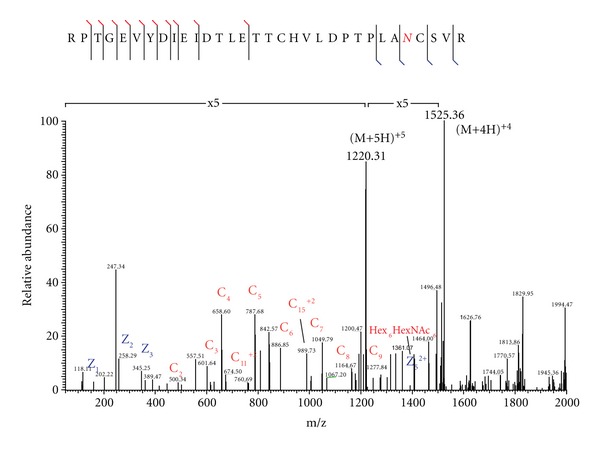
LC-MS ion trap ETD spectrum of bovine fetuin N-linked glycopeptide T72–103 precursor at m/z 1219.950 (5+).

To demonstrate the advantage of our novel acquisition strategy, we selected a twelve-protein mixture containing two glycoproteins: human serotransferrin and ovalbumin and contaminants of two others: bAGP and bovine fetuin. The mixture was digested with trypsin and analyzed by the traditional alternating HCD/ETD approach and the novel HCD-PD-ETD approach. Enrichment steps were avoided prior to analysis to ensure that the glycopeptides would be very low in abundances; this gave us the opportunity to evaluate the dynamic range of analysis for both acquisition strategies. Of the two, only HCD-PD-ETD approach identified glycopeptides from the contaminant glycoproteins (bAGP and bovine fetuin) present in the sample (Figure 2). While the HCD/ETD acquisition strategy identified numerous peptides and expected glycopeptides (eluted at 34, 40, and 48 min as shown in Figure 2 for HCD-PD-ETD approach) from serotransferrin and ovalbumin, it was unable to identify glycopeptides from the contaminant glycoproteins. Manual inspection of the RAW data confirmed this, as precursors associated with the contaminant glycopeptides were not targeted for MS/MS by HCD or ETD in the alternating approach. Analysis of the HCD-PD-ETD data revealed the identification of glycopeptides from expected glycoproteins and the contaminant glycoproteins. Identified minor glycopeptides from the contaminant glycoproteins by HCD-PD-ETD acquisition method are presented in Table 1, and representative ETD MS/MS spectra used in identification are shown in Figures 3 and 4. Because HCD-PD-ETD acquisition strategy did not waste time acquiring ETD data for nonglycosylated peptides, it was able to dig deeper into the sample and identify this low abundant glycopeptides that the alternating HCD/ETD acquisition strategy could not target.

In summary, we have been able to develop a novel acquisition strategy that minimizes some of the pain points associated with glycopeptide analysis. By combining the complementary information provided by HCD and ETD in an intelligent acquisition control, the overall productivity of glycopeptides analysis is improved.

## Figures and Tables

**Table 1:** List of identified contaminant glycopeptides and corresponding glycoforms from the 12-protein mixture digest by HCD-PD-ETD.

Peptide sequence	Glycan no.	Glycan composition
T103–109 Q*N*GTLSK(bAGP)	1	dHexHex_4_HexNAc_4_Neu5Gc_2_
	2	Hex_5_HexNAc_4_Neu5Gc_2_

T72–103 RPTGEVYDIEDTLETTCHVLDPTPLA*N*CSVR (bovine fetuin)	3	Hex_6_HexNAc_6_
	4	Hex_4_HexNAc_6_Neu5Ac
	5	Hex_5_HexNAc_5_Neu5Ac
